# Effective Fingerprint Quality Estimation for Diverse Capture Sensors

**DOI:** 10.3390/s100907896

**Published:** 2010-08-26

**Authors:** Shan Juan Xie, Sook Yoon, Jinwook Shin, Dong Sun Park

**Affiliations:** 1 Department of Electronics and Information Engineering, Chonbuk National University, 664-141 Ga Deokjin-Dong, Jeonju, Jeonbuk, 561-756, Korea; E-Mails: shanj_x@jbnu.ac.kr (S.J.X.); dspark@jbnu.ac.kr (D.S.P.); 2 Department of Multimedia Engineering, Mokpo National University, 61 Dorim-ri, Cheonggye-myeon, Jeonnam, 534-729, Korea; E-Mail: syoon@mokpo.ac.kr; 3 Advanced Graduate Education Center of Jeonbuk for EIT-BK21, Chonbuk National University, 664-141 Ga Deokjin-Dong, Jeonju, Jeonbuk, 561-756, Korea

**Keywords:** fingerprints, sensor, quality estimation, SVM, recognition

## Abstract

Recognizing the quality of fingerprints in advance can be beneficial for improving the performance of fingerprint recognition systems. The representative features to assess the quality of fingerprint images from different types of capture sensors are known to vary. In this paper, an effective quality estimation system that can be adapted for different types of capture sensors is designed by modifying and combining a set of features including orientation certainty, local orientation quality and consistency. The proposed system extracts basic features, and generates next level features which are applicable for various types of capture sensors. The system then uses the Support Vector Machine (SVM) classifier to determine whether or not an image should be accepted as input to the recognition system. The experimental results show that the proposed method can perform better than previous methods in terms of accuracy. In the meanwhile, the proposed method has an ability to eliminate residue images from the optical and capacitive sensors, and the coarse images from thermal sensors.

## 1. Introduction

Fingerprint recognition is the most widespread biometric authentication technology used in personal identification systems. The application of fingerprint recognition has been expanding owing to its uniqueness and security. Most available systems for fingerprint recognition use matching based on minutiae or local features of the fingerprint images [1]. It is well-known that these systems are very sensitive to noise or to quality degradation since the algorithms’ performance in terms of feature extraction and minutiae extraction generally relies on the quality of fingerprint images.

Bad-quality images mostly result in spurious and missing features that then degrade the performance of such systems. For many application systems, it is preferable to eliminate low-quality images and to replace them with acceptable higher-quality images to achieve better performance, rather than to attempt to enhance the first inputs. There have been studies on developing appropriate measures to assess the quality of fingerprint images for two types of fingerprint capture sensors – optical and capacitive sensors [2].

Representative quality assessment measures for images from various types of capture sensors have been known to vary due to the physical differences between the sensors. The optical sensor and capacitive sensor are easily affected by an unclean surface, and the residue fingerprint images can easily affect fingerprint quality. On the other hand, the thermal sensor is easily affected by the temperature and the coarse fingerprint image. Alonso-Fernandez *et al.* [2] investigated the relationship between sensor types and quality measures. They reveal that an excellent measure for optical sensor images could be the worst for those of capacitive sensors. Therefore, adaptive measures are required for images captured by various types of sensors, and this may be a major disadvantage in designing a general high-performance fingerprint recognition system.

In this paper, we develop an effective quality estimation system that can be used as a general quality estimation system for images from various types of sensors. The main idea of this paper is based on modifying the representative quality measures of one sensor to make them appropriate for other types of sensors, and to then estimate the quality of fingerprint input into three classes using the features extracted from the modified measures.

In order to evaluate the performance of the proposed system in terms of classification accuracy, 5,040 images from optical sensors, capacitive sensors and thermal sensors are tested. The performance of the proposed system with multiple features is compared with those of systems with single quality assessment measures.

The rest of the paper is organized as follows: Section 2 introduces the theory behind diverse fingerprint capture sensors, and this section also reviews the representative quality measures along with the interesting relationships between sensor technologies. Based on these important relationships, we describe our improved features for the proposed system with their advantages in Section 3. Section 4 demonstrates the experimental results of the proposed system performed on three public databases, FVC2000, FCV2002 and FVC2004. Section 5 concludes this paper.

## 2. Fingerprint Sensor Technology and Representative Quality Assessment Measures

**Figure 1.**
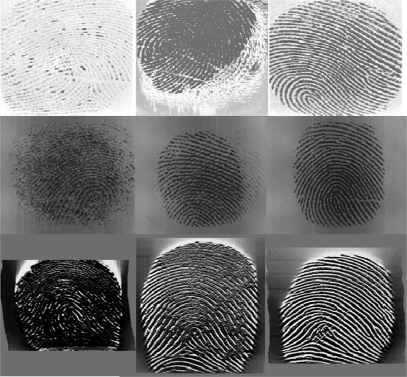
Fingerprint images from different capture sensors: (a) optical sensor, (b) capacitive sensor and (c) thermal sensor.

The quality of fingerprint images can vary depending on fingers conditions such as dried or moistened fingers, some exaggerated skin distortions and rotation, and acquisition sensors conditions such as fingerprint capture vertical position, pressures against the sensor surface, and temperature. Figure 1 shows examples of images representing three different quality conditions. The rows from top to bottom are captured by an optical sensor, capacitive sensor and thermal sensor. In each row, moving from left to right, the quality is bad, medium and good. Different factors affect diverse capture sensors.

For a fingerprint application system, identifying the quality of an input image in advance can be very crucial for improving system performance. By determining the quality of an input image, a system can either accept the current input as a valid one, or reject the input and acquire a new input.

### 2.1. Fingerprint Sensor Technology

The principle of the fingerprint acquisition process is based on geometric properties, biological characteristics and the physical properties of ridges and valleys [3]. The different characteristics obtained from ridges and valleys are used to reconstruct fingerprint images for different types of capture sensors.

- Geometry characteristics

The fingerprint geometry is characterized by protuberant ridges and sunken valleys. The intersection, connection and separation of ridges can generate a number of geometric patterns in fingerprints.

- Biological characteristics

The fingerprint biological characteristic means the ridge and valley have different conductivity, different dielectric constant of the air, different temperatures, and so on.

- Physical characteristics

Refering to the physical characteristics of the fingerprints, the ridges and valleys exert different pressures on the contact surface, and they have different pairs of wave impedance when they are focused on the horizontal plane.

According to these characteristics, there are two methods of capturing fingerprints. One type of sensors initially sends a detecting signal to the fingerprint, and it then analyzes the feedback signal to form a fingerprint ridge and valley pattern. Optical collection and Radio Frequency (RF) collection are two typical active collection sensors. Other fingerprint sensors are the passive ones. As the finger is placed on the fingerprint device, because of the physical or biological characteristics of the fingerprint ridges and valleys, the different sensors form different signals, and a sensor signal value is then analyzed to form a fingerprint pattern, such as in the thermal sensors, semiconductor capacitors sensors and semiconductor pressure sensors.

As optical sensors are based on the light reflection properties [2], which strictly impact the related gray level values, so that the gray level features-based measure quality, so Local Clarity Score ranks first for optical sensors. Optical sensors only scan the surface of the skin, not penetrating the deep skin layer. In case that there are some left over spots or trace from the previous acquisition of fingerprints, the resulting fingerprint may become very noisy resulting in difficulty in determining dominant ridges and orientations. This, in turn, makes the orientation certainty level of the fingerprint lower than that of a normal one.

A capacitive sensor uses the capacitance, which exists between any two conductive surfaces within some reasonable proximity, to acquire fingerprint images. The capacitance reflects changes in the distance between the surfaces [4]. The orientation certainty ranks first for the capacitive sensor since capacitive sensors are sensitive to the gradient changes of ridges and valleys.

A thermal sensor is made of some pyroelectric material that generates current based on temperature differentials between ridges and valleys. The temperature differentials produce an image when the contact occurs since the thermal equilibrium is quickly reached and the pixel temperature is stabilized. However, for the sweeping thermal sensor, the equilibrium is broken as the ridges and valleys touch the sensor alternately. Some parts of the fingerprint look coarse and have poor connectivity properties.

### 2.2. Representative Quality Estimation Measures

In previous studies [5–12], some fingerprint quality assessments have been performed by measuring features such as ridge strength, ridge continuity, ridge directionality, ridge-valley structure or estimated verification performance. Various types of quality measures have been developed to estimate the quality of fingerprints based on these features. Table 1 shows typical quality measures that have appeared in previous works. A high-quality image normally contains clear structural information with very low noise, so that we can easily distinguish ridges and precisely locate most of the minutiae. On the other hand, a low-quality fingerprint image often has blurred ridges and valleys, and henceforth a single basic feature extracted from the image can be erroneous.

Some interesting relationships between capture sensors and quality measure have been found in [2]. Orientation Certainly Level (OCL) and Local Orientation Quality (LOQ) measures that rely on ridge strength or ridge continuity perform best in capacitive sensors, while they are the two worst quality measures for optical sensors. The gray value based measures rank first for optical sensors as they are based on light reflection properties that strictly impact the related gray level values repetitive (2.1) Our proposed method is not only based on the basic fingerprint properties, but also on the physical properties of the various sensors.

## 3. Proposed Features for Estimation System

Quality assessment measures can be directly used to classify input fingerprints of a quality estimation system. The discrimination performance of quality measures, however, can be significantly different depending on the sensors and noise sources. To construct a general estimation system that can be adaptable for various input conditions, in this paper, we generate a set of features based on the analysis of quality measures.

**Figure 2.**
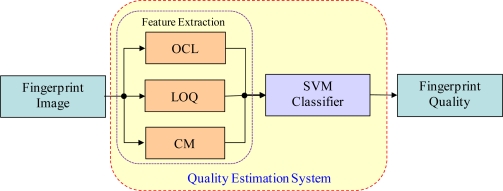
Fingerprint image quality estimation system.

Figure 2 shows the overall block diagram of the proposed estimation system. From the analysis of various quality measures of optical sensors, capacitive sensors and thermal sensors, Orientation Certainty, Local Orientation Quality and Consistency are selected for generating the features of the proposed system. The orientation certainty and local orientation quality measures are the two best measures for capacitive sensors; moreover, in this study, we develop highly improved features from these measures, along with the consistency measure, for images obtained from optical sensors and thermal sensors. The extracted features are then used to classify an input image into three classes, Good, Medium and Bad quality, using the well-known support vector machine (SVM) [13] as the classifier.

Optical sensors are widely used for their reliability and durability; capacitive sensors have become popular due to their high quality images, and thermal sweep sensors hold an extensive market segment. In our attempt, two best/worst measures for capacitive/optical sensors, OCL and LOQ, are used to assess images obtained from optical sensors with much higher performance. We design and extract features from the two measures along with another measure for assessing consistency. Using the combination of the features, the proposed estimation system classifies input images from any type of sensors.

### 3.1. Orientation Certainty Calculation

To estimate the orientation certainty for local quality analysis, we partition the fingerprint image into non-overlapping blocks with a size of 32 × 32 pixels [6,8,11,14,15]. A covariance matrix of the gradient vector is contributed to estimate the orientation certainty. The covariance matrix that is constructed by *C* of the gradient vector for an *N* points image block can be expressed as in Equation (1):

(1) $$C=\frac{1}{N}\sum_{N}\left\{\left[\begin{array}{l} \mathrm{dx}\\ \mathrm{dy} \end{array}\right]\left[\begin{array}{ll} \mathrm{dx} & \mathrm{dy} \end{array}\right]\right\}=\left[\begin{array}{cc} c_{\mathrm{xx}} & c_{\mathrm{xy}}\\ c_{\mathrm{yx}} & c_{\mathrm{yy}} \end{array}\right]$$

In this equation, *dx* and *dy* represent the intensity gradient of each pixel calculated by Sobel operator. The orientation certainty of the block can be estimated by two eigenvalues of the covariance matrix.

The eigenvectors of *C* are called principal directions and are directions of pure curvature that are denoted *λ_a_* and *λ_b_*. The first eigenvector whose corresponding eigenvalue has the largest absolute value is the direction of the greatest curvature [16]. The other orthogonal eigenvector is the direction of least curvature. The corresponding eigenvalues are the respective amounts of these curvatures:

(2) $$\lambda_{a}=\frac{c_{\mathrm{xx}}+c_{\mathrm{yy}}+\sqrt{{\left(c_{\mathrm{xx}}-c_{\mathrm{yy}}\right)}^{2}+4c_{\mathrm{xy}}{}^{2}}}{2}$$

(3) $$\lambda_{b}=\frac{c_{\mathrm{xx}}+c_{\mathrm{yy}}-\sqrt{{\left(c_{\mathrm{xx}}-c_{\mathrm{yy}}\right)}^{2}+4c_{\mathrm{xy}}{}^{2}}}{2}$$

(4) $$\mathrm{Orientation}\_\mathrm{certainty}=1-\frac{\lambda_{b}}{\lambda_{a}}$$

Since *λ_a_* is greater than *λ_b_* in Equations (2) and (3), *λ_b_*/*λ_a_* is less than 1. When two eigenvalues, *λ_a_* and *λ_b_*, have similar magnitudes, namely there is not a strong orientation, *λ_b_*/*λ_a_* approaches to 1. However, when two eigenvalues have a very large gap between them, namely there is a strong orientation, then *λ_b_*/*λ_a_* approaches 0. Therefore, (1−*λ_b_*/*λ_a_*) in Equation (4) indicates the orientation certainty value from ‘1’ which represents a very strong orientation certainty to ‘0’ which represents a very weak orientation certainty due to no orientation or omnidirectional orientation.

**Figure 3.**
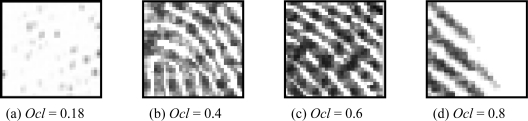
Fingerprint blocks with variable orientation certainty.

Figure 3 shows examples of blocks with four different orientation certainty values. Figure 3(a) has a low orientation certainty as the ridges in the block are omnidirectional. The ridges are not clear and also affected by some random noises. In Figure 3(b), the direction of the lower part is quite different from the upper part, while the ridges in Figures 3(c), (d) are parallel to each other. For a high certainty block, ridges and valleys are very clear with accordant orientation and, as the value decreases, the orientations change irregularly. The orientation certainty is introduced to describe how well the orientations over a neighborhood are consistent with the dominant orientation. It will not be affected by the edges.

In [8], the average of OCL values is used as feature for their estimation. To make the features more accurate, we introduce an optimization named “Pareto efficient” or “Pareto optimal” [14,15,17] to define four classes of blocks and use the normalized number of blocks as a feature for each class. The Pareto optimality is a concept from economics with applications in engineering and social sciences, which uses the marginal rate of substitution to optimize the multi-objectives. To obtain features from OCL values for the proposed system, we classify blocks into four different classes, from good to very poor, as in Table 2, by selecting three optimal thresholds*(x_1_, x_2_, x_3_)*. Three optimal threshold values are assumed to be located in the ranges shown in Equation (5) and selected by resolving the multi-object optimization. In Equation (5), we define the contrast covered by each class limited by optimal thresholds and find three thresholds that maximize the three areas at the same time:

(5) $$\{\begin{array}{ll} D_{1}=\int_{x_{0}}^{x_{1}}\left|\mathrm{gOclNum}(x)-\mathrm{bOclNum}(x)\right|dx & x_{0}\leq x\leq x_{1}\\ D_{2}=\int_{x_{1}}^{x_{2}}\left|\mathrm{gOclNum}(x)-\mathrm{bOclNum}(x)\right|dx & x_{1}<x\leq x_{2}\\ D_{3}=\int_{x_{2}}^{x_{3}}\left|\mathrm{gOclNum}(x)-\mathrm{bOclNum}(x)\right|dx & x_{2}<x\leq x_{3}\\ D_{4}=\int_{x_{3}}^{x_{4}}\left|\mathrm{gOclNum}(x)-\mathrm{bOclNum}(x)\right|dx & x_{3}<x\leq x_{4} \end{array}$$

In this equation, *gOclNum(x)/bOclNum(x)* represents the number of blocks when the OCL value equals *x* from the good/bad-quality image. *D_i_* represents the contrast of a level between good and bad quality. Obviously, if the contrast becomes larger, then it becomes easier to classify with a higher classification rate.

Three thresholds, (*x_1_, x_2_, x_3_*) grade the blocks into four classes. We can get the optimal value for (*x_1_, x_2_, x_3_*) by Equation (5). Through the optimization process, we obtain three thresholds, *x_1_* = 0.01, *x_2_* = 0.4, *x_3_* = 0.8, along with *x_0_* = 0, *x_4_* = 1. As in Table 2, we define four classes of blocks according to their OCL values. The OCL features of the estimation system are then defined as the normalized amount of blocks for each class as shown in Equation (6):

(6) $$\mathrm{feature}\_i=\frac{\int {}_{x_{i-1}}^{x_{i}}\mathrm{OclNum}(x)dx}{\mathrm{BlockNum}}(1\leq i\leq 4)$$

**Figure 4.**
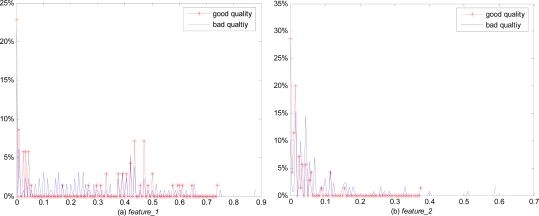

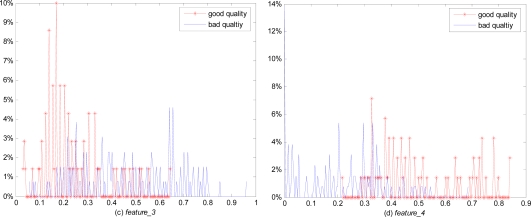
The distribution of four optical sensor features.

In Equation (6), *OclNum*(*x*) represents the number of blocks whose OCL values are equal to *x. BlockNum=Imagesize/Blocksize* and the sum of these four features becomes unity. Figure 4 shows the distribution of *features_1–4* of fingerprint captured by the optical sensor in FVC2004 database. We can infer an obvious tendency that good quality images have relatively larger values for *feature_4* than bad quality images, while they have smaller values for *feature_1 and feature_2*. Three independent features: *feature_2*, *feature_3* and *feature_4* are contributed to the three input vectors for the classification. And *feature_1* which represents the ratio of the background is useful for the estimation of fingerprint local orientation quality.

### 3.2. Consistency Measure (CM)

For the thermal sensor, its equilibrium is broken as the ridges and valleys touch the sensor alternately and is also affected by the environmental temperature. Sometimes fingerprints captured by it are coarse, as shown in Figure 1. Different from bad quality images from optical and capacitive sensors, a poor thermal sensor image still has a good orientation, and its ridges and valleys are still clearly separate. However, the consistency of the bad part obviously performs worse than the good quality one.

In addition, when optical and capacitive sensors are used, a bad quality image often carries broken ridges or valley regions due to the residue from previous data acquisitions or low pressure against the sensor surface. However, in a good quality image, ridges or valley regions are fairly consistent.

The consistency measure proposed in [7] calculates the consistency of ridges and it was used as a feature to represent the overall consistency of an image. However, noises affect not only ridges but also valleys. In order to minimize the effect of noises, we propose to use the consistency of both ridges and valleys to represent the overall consistency of an image.

To measure the consistency of an image, an image is binarized with optimum threshold values obtained from the Otsu method [18]. The consistency in a pixel position is calculated by scanning the binary image with a 3 × 3 window by two cases as shown in Equation (7). When the scanned block is the valley part, the sum value of the binary block would be between [0,4]. Another case is prepared for the ridges with the sum values are between [5,9]. Both the higher values are contributed to high consistency as the neighborhood pixels have the same state as that of the center pixel. And a lower value indicates a randomly organized structure and hence lower quality. The feature for the consistency of an input image can be averaged as in Equation (8):

(7) $$\mathrm{con}(i,j)=\{\begin{array}{ll} 0.2\cdot (8-\mathrm{sum}(i,j))\cdot (1-c(i,j))+c(i,j) & 4<\mathrm{sum}(i,j)\leq 8\\ 0.2\cdot \mathrm{sum}(i,j)\cdot c(i,j)+(1-c(i,j)) & 0\leq \mathrm{sum}(i,j)\leq 4 \end{array}$$

(8) $$\mathrm{feature}\_5=\frac{\sum_{m}\sum_{n}\mathrm{con}(m,n)}{\mathrm{Num}}\;(\mathrm{for}\;m,n=2,5,\cdots ,3k+2,\cdots ,255);$$

In these equations, *Num* = *ImageSize*/*NeighborSize* which represent the size of input image and the size of the neighborhood. *c(i,j)* represents the binary value of the center pixel and *sum(i,j) is* the sum of binary values of its 3 × 3 neighborhood, respectively.

### 3.3. Local Orientation Quality (LOQ)

A high-quality image contains very clear local orientations, and measuring the curvature of such images with local orientations can be used to precisely find a core point region and invalid curvatures. The LOQ is a quality assessment measure based on local orientations [7] as described in Steps 1–3.

#### Step 1:

**Figure 5.**
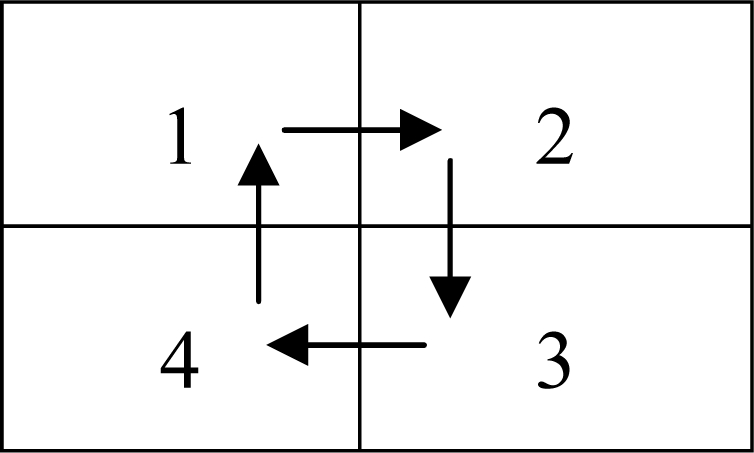
Quadrants of the Original LOQ measure.

Partition each sub-block into four quadrants as shown in Figure 5 and compute the absolute orientation difference of these four quadrants in clockwise direction. The absolute orientation difference is typically greater than zero since the orientation flow in a block is gradually changed.

#### Step 2:

Calculate the local orientation quality for each block. As shown in Equation (9), if the absolute orientation change is larger than a certain value, in this case, 8-degrees, then we assume that the block contains an invalid curvature change of quadrants. The sum of the four quadrants is determined as the local orientation quality of the block:

(9) $$O_{\mathrm{mn}}=\{\begin{array}{cc} 0 & \left|\mathrm{ori}(m)-\mathrm{ori}(n)\right|\leq 8{}^{\circ}\\ 1 & \left|\mathrm{ori}(m)-\mathrm{ori}(n)\right|>8{}^{\circ} \end{array}$$

(10) $${\mathrm{loq}}_{1}(i,j)=O_{12}+O_{23}+O_{34}+O_{41}$$

In the equation, *ori(m)* denotes the orientation value of quadrant *m*.

#### Step 3:

Compute the preliminary local orientation quality of the fingerprint image.

The LOQ value of an image is then computed as the total angular change of blocks with M×N blocks in Equation (11):

(11) $${\mathrm{LOQ}}_{1}=\sum_{i=1}^{M}\sum_{j=1}^{N}{\mathrm{loq}}_{1}(i,j)$$

This preliminary local orientation quality of the fingerprint may include some false positives due to the light reflection properties of optical sensors and the orientation calculation based on gray-level values. To supplement the artifact, we design additional steps as below.

#### Step 4:

Compute the new local orientation quality of the quadrants.

Based on Step 1, we label these block quadrants whose orientation change is more than 8 degrees. We can see the block orientation changes only in two directions: horizontal and vertical.

**Figure 6.**
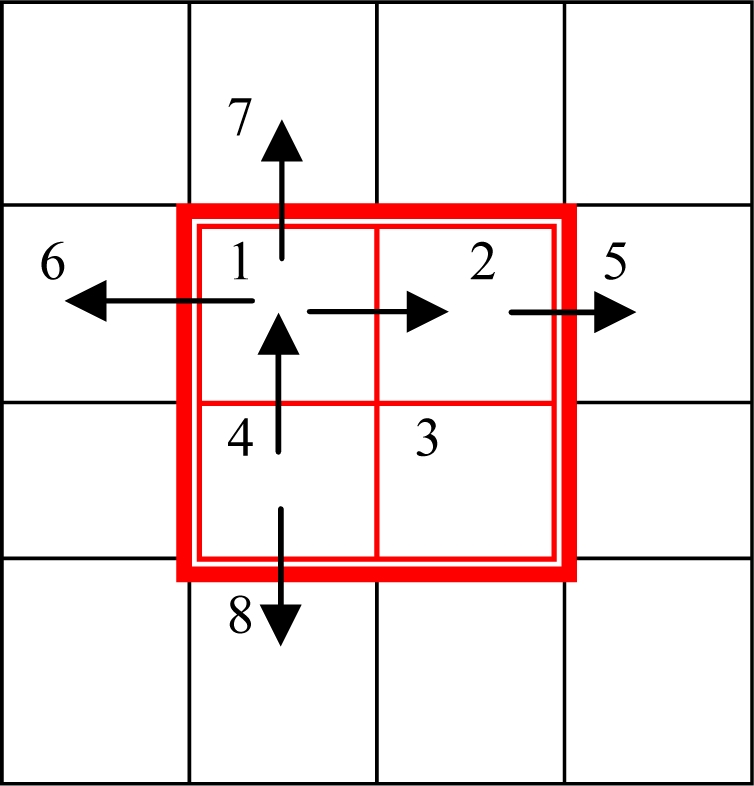
The basic idea of the LOQ measure.

As shown in Figure 6, if quadrant 1 and 2 are labeled for their horizontal orientation change, then we detected their horizontal neighbor quadrants. That means we calculate *O_16_* and *O_25_* in Equation (9), which are the orientation change from quadrant 1 to quadrant 6 and quadrant 2 to quadrant 5, respectively.

If we detect vertical change in Step 2, then we need to calculate *O_17_* and *O_48_* in Equation (9), which are the orientation change from quadrant 1 to quadrant 7 and quadrant 4 to quadrant 8, respectively. We can set a special label for each detected quadrant to avoid repeating detection. The amount of new invalid curvature blocks are set as *LOQ_2_*. Then, we can get *LOQ_2_* by the sum of the *loq_2_(i, j)* of unrepeated detection quadrants *(i, j)*:

(12) $$\mathrm{lo}q_{2}(i,j)=(O_{25}+O_{16})\cdot \mathrm{Horizontal}+(O_{17}+O_{48})\cdot \mathrm{Vertical}$$

(13) $${\mathrm{LOQ}}_{2}=\sum_{i=1}^{M}\sum_{j=1}^{N}\mathrm{lo}q_{2}(i,j)$$

If there is an orientation change in horizontal, then Horizontal = 1, otherwise, Horizontal = 0. Vertical is same as Horizontal.

#### Step 5:

*LOQ_1_* indicates the invalid orientation change of each local block itself, while *LOQ_2_* means the invalid orientation change between a block and another.

Using *LOQ_1_* and *LOQ_2_*, *feature_6* is defined as follows:

(14) $$\mathrm{feature}\_6=\frac{{\mathrm{LOQ}}_{1}+{\mathrm{LOQ}}_{2}}{4\times (\mathrm{BlockNum}-\mathrm{Num}(\mathrm{OCL}=0))}$$

Where, *BlockNum = Imagesize/Blocksize*, *Num (Ocl =* *0)* express the amount of background blocks which is approximately computed by *Num (Ocl =* *0)* ≈ *BlockNum* × *feature*_1.

Each sub-block is partitioned into 4 quadrants. In this paper, the image size is 256 × 256, and the block size is 32 × 32, and thus *BlockNum = 64*.

### 3.4. Classifier

The SVM is a powerful classifier with an excellent generalization capability that provides a linear separation in an augmented space by means of different kernels [13]. The kernels map input data vectors onto a high-dimensional space where a linear separation is more likely, and this process amounts to finding a non-linear frontier in the original input space.

Each input vector for the proposed quality estimation system consists of five features as in Equation (15).

(15) V=[feature_2,feature_3,feature_4,feature_5,feature_6]

*feature_2, feature_3 and feature_4* are three independent features chosen from four features related to the OCL measure, defined in Equation (6), representing the normalized amounts of blocks for each grade. *feature_5* stands for the overall consistency, and *feature_6* is the average LOQ computed from the number of blocks with invalid direction changes.

## 4. Experimental Results

### 4.1. Database Description

To evaluate the performance of the proposed quality estimation system, three public databases FVC2004, FVC2002, FVC2000 [19–21] are used. The three databases include several sets of fingerprints from various sensors. Table 3 shows the detail information of fingerprint databases. There are 80 images in each Set_B database and 800 images in Set_A database. Each image is divided into 64 blocks, each with the size of 32 × 32 pixels. Although the types of sensor are adopted in the database, the basis acquisition physical principle is same for all optical, capacitive and thermal sensors.

### 4.2. Classification of Fingerprints in the Database According to Their Quality

**Figure 7.**
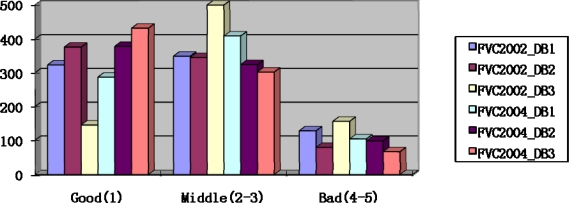
Quality distribution of the databases by the relabeled NFIS method.

The NFIS method [9,10] is the most widely used method for fingerprint quality estimation. The method proposed the assumption that fingerprint quality is a predictor of matcher performance. A good quality image will result in a high matcher performance, while a bad quality image will be easily rejected. The method computes several features including direction map, low contrast, low flow and high curve to classify a fingerprint into one of five levels of quality. We redivide the NFIS quality ratings from five levels into three levels, where level 1 belongs to the Good class, levels 2–3 belong to the Medium class and levels 4–5 are the Bad class. Figure 7 shows the quality distribution of FVC2002 and FVC2004 by the relabeled NFIS method. As shown in Figure 7, there are the most Good quality fingerprints in the database FVC2004_DB3 captured by thermal sensors, while the FVC2002_DB3 database captured by the capacitive sensor includes the least number of Good quality fingerprints. Each fingerprint assigned to a class according to the NFIS quality reclassified to three classes is used to verify the proposed method.

### 4.3. Quality Estimation Performance

In the experiment, we apply the 10% Jackknife procedure by using 90% of the images for training and 10% for testing, respectively. Four different kernels, linear, polynomial, RBF and sigmoid, are implemented for the SVM classifier to investigate the performance with different classifier conditions.

Table 4 shows the classification accuracy rate of the original OCL, CM, LOQ measures and their improved versions when they are used separately as a single quality measure. They are the result of the simulation where the SVM classifier uses the RBF kernel, which shows better results than other kernels. In comparison with the original average OCL measure, the proposed OCL measure achieves better results by adding the optimal determining system which detects not only the local orientation stabilities but also the global ones. Moreover, for the LOQ measure, accuracy is increased after adding the additional orientation step.

From Table 5, we can find that the accuracy increases by combining quality estimation measures. The OCL, CM and LOQ features represent different characteristics of the fingerprint. The OCL feature measures the orientation stability of the ridge. The CM feature involves the ridge connection and can detect small noises, while the LOQ feature identifies the irregular direction change of ridges. These different measures can make up for each other and thus give better results.

As shown in Table 5, the accuracy rates of the proposed combined measure are 95.62%, 95.50% and 96.25%, respectively, for the optical, capacitive and thermal sensor. Compared to the NFIS method, our proposed method achieves high accuracy with fewer features. In addition, since our proposed method didn’t need to detect fingerprint minutiae before the quality estimation, it has considerably less computational complexity than the NFIS method.

**Figure 8.**
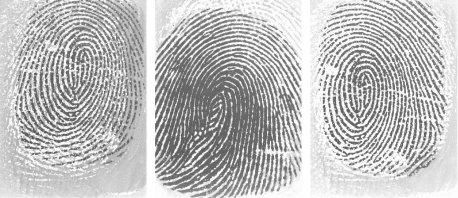
Examples of the residue images captured by optical sensors.

Figure 8 shows three residue images captured by optical sensors. All of these fingerprints are evaluated as best quality by the NFIS method which classifies the fingerprint into five classes rated from 1 to 5. As residue fingerprints appear frequently in the database from the optical sensors, the problem that residue images are considered as fingerprints with the best quality cannot be ignored. In the database FVC2004 DB1_A and FVC2004 DB2_A, there are about 82 images with obvious residue. We estimate the image quality both by our proposed method and the NFIS method. The comparative results are shown in the Table 6. The error rate of our proposed method is 3.65%, while the error rate of NFIS method is 12.20%. The NFIS method mistakes the prior image as the minutiae of the remained fingerprint. The proposed system, however, can avoid this kind of residue mistaken error via the global orientation certainty.

**Figure 9.**
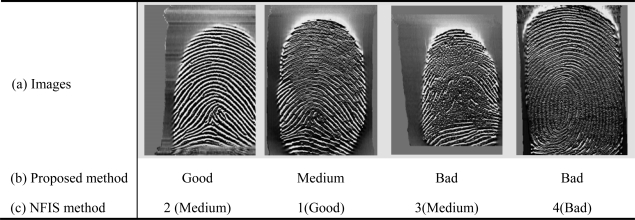
Examples of images captured by thermal sensors with different roughness and their results obtained from two different quality estimation methods.

Figure 9 (a) shows examples of images captured by thermal sensors but with different roughness. The left one is prominently smoother than the other three images. Due to the fact the thermal sensor is sensitive to the temperature, the image looks coarse when the finger temperature is near to the environment. By using the combination of the fingerprint consistency and orientation certainty as a feature for the quality estimation, the coarse image with bad quality can be selected out. Figures 9 (b) and (c) show the results obtained from two different quality estimation methods using our proposed method and NFIS method, which are applied to each image of Figure 9 (a). An image of the first column is of lower quality than an image of the second one, but it is found that the NFIS method ignores the roughness of images.

Both the residue image from the optical or capacitive senor and the coarse image from the thermal sensor will affect the fingerprint matching performance. Therefore, by eliminating these kinds of bad quality images, fingerprint matching performance will be improved.

## 5. Conclusions

Since fingerprints have different characteristics according to the sensor technology used to acquire them, the selection of features for fingerprint quality measurements is closely related to the sensors used. Moreover, there is no feature which performs well for all the different types of sensors. In this paper, we considered three kinds of sensors—optical, capacitive, and thermal sensors—as capture sensors and three features, OCL, CM and LOQ, commonly used in the fingerprint estimation. First, we have proposed an improved method of estimating the quality of existing fingerprints using these features individually and verified the improvements through simulation. Then a combinational method was proposed for fingerprint quality estimation for diverse capture sensors and it was verified that it is effective through the simulation. The experimental results showed that the proposed quality estimation system can perform better than previous methods in terms of accuracy for different capture sensors. The Orientation Certainty feature of optical sensors is more effectively extracted by using the proposed quality measure. In case of the Local Orientation quality, by enlarging the detection region along the changed direction, accuracy has been improved by about 5% for the three kinds of capture sensors. The consistency measure has been found to achieve good performance for thermal sensors. When using the proposed series of measures, accuracy is 95.62%, 95.50%, 96.25% for the optical, capacitive and thermal sensor, respectively. Compared to other quality estimation measures, the improvements are not only beneficial to accuracy, but also to eliminating residue images from the optical and capacitive sensor, and the coarse images from the thermal sensor. Eliminating both the residue images and coarse images improves the fingerprint matching performance.

## Figures and Tables

**Table 1.** Summary of Representative Quality Measures.

**Local Features-based**	OCL: Orientation certainty level FREC: Ridge frequency, ridge thickness, ridge-to-valley thickness. LOQ: Local orientation quality LCS: Local clarity score
**Power Spectrum-based**	Energy: Energy concentration in ring-shaped regions of the spectrum.
**Classifier-based**	NFIS: Matcher performance, use the degree of separation between the match and non-match distributions

**Table 2.** Classification levels of the orientation certainty value.

**OCL Range**	**Classification Level**

0.8 ≤ OCL ≤ 1	Good block
0.4 ≤ OCL < 0.8	Normal block
0.01 ≤OCL< 0.4	Bad block
0 ≤ OCL < 0.01	Very bad block or background

**Table 3.** Information on the capture sensor of the database.

	**Optical sensor**	**Capacitive sensor**	**Thermal sensor**
FVC2000	DB1_B, DB3_B	DB2_B	-
FVC2002	DB1_A,DB2_A	DB3_A	-
FVC2004	DB1_A,DB2_A	-	DB3_A

**Table 4.** Comparison of the classification accuracy rate when a single quality measure is used.

	**Original method**			**Proposed method**		

	OCL	CM	LOQ	OCL	CM	LOQ
Optical	80.05%	81.68%	77.86%	87.50%	81.99%	81.86%
Capacitive	83.18%	74.62%	87.79%	90.56%	81.61%	89.10%
Thermal	78.84%	77.90%	78.72%	81.96%	89.42%	83.04%

**Table 5.** Comparison of the classification accuracy rate when combined quality measures are used.

	**OCL + CM**	**CM + LOQ**	**LOQ + OCL**	**LOQ + OCL + CM**
Optical	92.62%	91.25%	91.00%	95.62%
Capacitive	93.25%	91.88%	92.38%	95.50%
Thermal	94.00%	93.95%	86.14%	96.25%

**Table 6.** Classification results of residue images by the proposed method and NFIS method.

		**Proposed method**			**NFIS method**		

		**Good**	**Medium**	**Bad**	**Good (1)**	**Medium (2–3)**	**Bad (4–5)**
**Subjective Quality**	**Good**	44	1	0	43	2	0
	**Medium**	2	21	0	4	19	0
	**Bad**	0	0	14	2	2	10

## References

[1] Yang JC, Shin JW, Min BJ, Park JB, Park DS (2006). Fingerprint Matching Using Invariant Moment FingerCode and Learning Vector Quantization Neural Network. Comput. Intell. Security.

[2] Alonso-Fernandez F, Fabio R, Fierrez J, Ortega-Garcia J (2007). Comparison of fingerprint quality measures using an optical and a capacitive sensor.

[3] Maltoni D, Maio D, Jain AK, Prabhakar S (2009). Handbook of Fingerprint Recognition.

[4] Overview of Capacitive Sensors. http://www.lionprecision.com/capacitive-sensors/index.html.

[5] Alonso-Fernandez F, Fierrez J, Ortega-Garcia J, Gonzalez-Rodriguez J, Fronthaler H, Kollreider K, Bigun J (2007). A Comparative Study of Fingerprint Image-Quality Estimation Methods. IEEE Trans. Inf. Foren. Sec.

[6] Lim E, Jiang XD, Yau WY Fingerprint Quality and Validity Analysis.

[7] Lim E, Toh KA, Suganthan PN, Jiang XD, Yau WY Fingerprint Image Quality Analysis.

[8] Chen T, Jiang X, Yau W Fingerprint Image Quality Analysis.

[9] Tabassi E, Wilson C, Watson C (2004). Fingerprint Image Quality, NIST research report NISTIR7151.

[10] Tabassi E, Wilson C A Novel Approach to Fingerprint Image Quality.

[11] Wu J, Xie SJ, Song DH, Lee WD A New Approach for Classification of Fingerprint Image Quality.

[12] Lee SH, Lee CH, Kim JH (2005). Model-based quality estimation of fingerprint images. Lect. Note. Comput. Sci.

[13] Suykens JAK (2001). Nonlinear Modeling and Support Vector Machines.

[14] Xie SJ, Yang JC, Yoon S, Park DS (2008). An Optimal Orientation Certainty Level Approach for Fingerprint Quality Estimation.

[15] Xie SJ, Yoon S, Yang JC, Park DS Rule-based Fingerprint Quality Estimation System Using the Optimal Orientation Certainty Level Approach.

[16] Ando S (2000). Image field categorization and edge/corner detection from gradient covariance. IEEE Trans. Patt. Anal. Mach. Int.

[17] Obayashi S, Sasaki D, Oyama A (2004). Finding Tradeoffs by Using Multi-Objective Optimization Algorithms. Trans. Jpn. Son. Aeronaut. Space Sci.

[18] Ostu N (1979). A Threshold Selection Method from Gray-Level Histograms. IEEE Trans. Syst.

[19] FVC 2000 Fingerprint Verification. http://bias.csr.unibo.it/fvc2000/databases.asp.

[20] FVC 2002 Fingerprint Verification. http://bias.csr.unibo.it/fvc2002/databases.asp.

[21] FVC 2004 Fingerprint Verification. http://bias.csr.unibo.it/fvc2004/databases.asp.

